# Cocaine Sniffing

**Published:** 1900-12

**Authors:** 


### "COCAINE SNIFFING."

ONE of the most pathetic instances of the negro's inability to resist the vices of civilization is offered by his ever-increasing addiction to the cocaine habit. In this city cocaine as a means of intoxication is actually replacing alcohol among the colored "boulevardiers" and their female associates of Decatur street. Parties and clubs are organized for bacchanalian indulgence in the drug, in which often as many as a hundred persons participate. A room corresponding to the "gin-mill" or the opium joint is supplied to his patrons by the seller of the drug, where they take it without fear of molestation or interruption. There are said to be two such places on Decatur street being operated in connection with drug stores, and that the proprietors are getting rich in the awful traffic. Ten cents buys four grains of cocaine and permission to use the room.

The drug is sniffed up the nose, and the habitué lolls at ease and gives himself over unreservedly to its influence. Young men, young women and boys constitute the bulk of the victims of an enslavement far exceeding that which led to the bloodiest war of modern times, for it not only brings the body under its grim subjection, but warps and perverts the mental and moral nature almost beyond the hope of redemption. Could a more ideal victim be found than the negro, "half devil and half child," and one with whom the cocaine habit could play more rapid, more complete and utter havoc?

On Saturday nights, and then on until the week's earnings are expended, these resorts number their patrons by the hundreds, some of them novices, others hollow-eyed, trembling wrecks, upon whom the drug has obtained a hold that cannot be broken. The latter, in the vernacular, are known as cocaine "sniffers," and it is needless to say are in the majority. It is said that these facts are well known to the police, but, as there is a superficial form of observance of the legal restrictions upon the sale of poisons, they are powerless to suppress the traffic. It is inconceivable that any one could be so sunken in moral debasement as not only to pander to a vicious, perverted and, in the highest degree, destructive taste, but could lend his aid in leading the uninitiated and uninformed into the path that kills. Yet all this can be charged to those purveyors of the drug who are willing to accept any flimsy pretext to supply their customers with that which their warped nature craves.

Already the negro problem is the incubus that the South must unwillingly bear, and it is not short of criminal to complicate it by artificially disturbing a nature which even normally is with difficulty restrained. Nothing but the most severe and drastic measures are competent to correct this growing and threatening evil, and it is hoped that once the attention of the proper authorities is directed at it, the monstrous offense will be punished by suppression.

